# The Good, the Bad, and the Tiny: A Simple, Mechanistic-Probabilistic Model of Virus-Nutrient Colimitation in Microbes

**DOI:** 10.1371/journal.pone.0143299

**Published:** 2015-11-23

**Authors:** B. B. Cael

**Affiliations:** 1 Massachusetts Institute of Technology, Cambridge, MA, United States of America; 2 Woods Hole Oceanographic Institution, Woods Hole, MA, United States of America; University of Malaya, MALAYSIA

## Abstract

For phytoplankton and other microbes, nutrient receptors are often the passages through which viruses invade. This presents a bottom-up vs. top-down, co-limitation scenario; how do these would-be-hosts balance minimizing viral susceptibility with maximizing uptake of limiting nutrient(s)? This question has been addressed in the biological literature on evolutionary timescales for populations, but a shorter timescale, mechanistic perspective is lacking, and marine viral literature suggests the strong influence of additional factors, e.g. host size; while the literature on both nutrient uptake and host-virus interactions is expansive, their intersection, of ubiquitous relevance to marine environments, is understudied. I present a simple, mechanistic model from first principles to analyze the effect of this co-limitation scenario on individual growth, which suggests that in environments with high risk of viral invasion or spatial/temporal heterogeneity, an individual host’s growth rate may be optimized with respect to receptor coverage, producing top-down selective pressure on short timescales. The model has general applicability, is suggestive of hypotheses for empirical exploration, and can be extended to theoretical studies of more complex behaviors and systems.

## Introduction

Phytoplankton account for approximately half of global annual primary production [1]; both phytoplankton and marine bacteria are important components in global biogeochemical cycles and the global ecosystem [2]. Though phytoplankton and other marine microorganisms are regulated simultaneously by bottom-up [3, 4] and top-down control [5], the former frequently a result of nutrient limitation, the latter frequently a result of viral invasion [6], these controls are seldom studied simultaneously. Both grazing effects and nutrient limitation are well-studied [7, 8]; understanding of viral impacts remains poorer [9] but viruses are known to have significant effects on a vast range of marine microbes; studies include e.g. estimates that 30% of cyanobacterial death is caused by viral lysis [10], or laboratory results showing a 20% increase in viral concentration can halve phytoplankton primary productivity and biomass [11]. Viral invasion also plays an important role in biogeochemical cycles through the “viral shunt” [12]. Viruses often occur at an order of magnitude higher concentration in marine environments than even prokaryotes, [13], up to 10^9^ml^−1^ in some cases. These estimates are given for total number of viruses; however, the networks of virus-host interactions are complex, and involve both specialization and generalization [14, 15, 16].

Viruses typically inject their genetic material into hosts through specific channels on the cell surface [17]; some of these channels in both phytoplankton and bacteria are the receptors through which the organism takes up nutrients [18, 19, 20]. This presents an interesting co-limitation scenario, where the would-be-host must balance minimizing viral susceptibility with maximizing uptake of limiting nutrient(s). This question was deftly addressed on evolutionary timescales using adaptive dynamics in a 2009 paper by Menge and Weitz [21]. However, there are complicating factors on longer timescales, such as the co-evolving process of ‘lock and key’ dynamics between host and virus [22]; simultaneously, some microorganisms have been shown to express control over their receptor availability on short timescales [23, 24], and the dynamics of encounter rates are nontrivial and may yield significantly different results than a prescribed viral encounter rate. Classicaly in marine systems, viral uptake has been modeled as a rate process [25], but as marine viruses are often quite virulent [6, 17] and their invasion-to-lysing timescales are typically much shorter than host replication rates [26, 27], modeling viral invasion in this way is not faithful to the system considered herein (this paper focuses on marine environments). Instead, to garner a robust, mechanistic understanding of the dynamics of viral invasion, it must be modeled probabilistically as a first-passage-time process, based on the Brownian motion of the viruses, and by comparing timescales of nutrient uptake-limited host reproduction with viral invasion times. This is to say that hosts do not accumulate viruses at a particular diffusive rate; once a host has been invaded, the virus quickly dominates its replicative machinery, and its lysing is more or less assured.

In what follows I develop a simple mechanistic model from first principles to address this co-limitation on shorter timescales, from an individual host’s perspective. It should not be considered a thorough explication of the relevant factors’ effect on individual growth rate, but rather a hypothesis-generating tool for laboratory or *in situ* investigation, suggesting among other things i) that an individual’s lineage’s growth rate may indeed be optimized with respect to receptor coverage, on various timescales, but only in a restricted set of conditions where viruses are plentiful and/or hosts are aggregated, ii) which conditions might be expected to result in a microbial population being drawn down to a seed population by phage activity, iii) that organisms with fast controls on their receptor activity might be advantaged by reducing receptor activity in host-aggregated or high-viral conditions, iv) that intermittency in viral concentration might serve as a means to support phenotypic diversity in a microbial population in terms of receptor allocation.

## Analysis

### Setup

**Fig 1:**
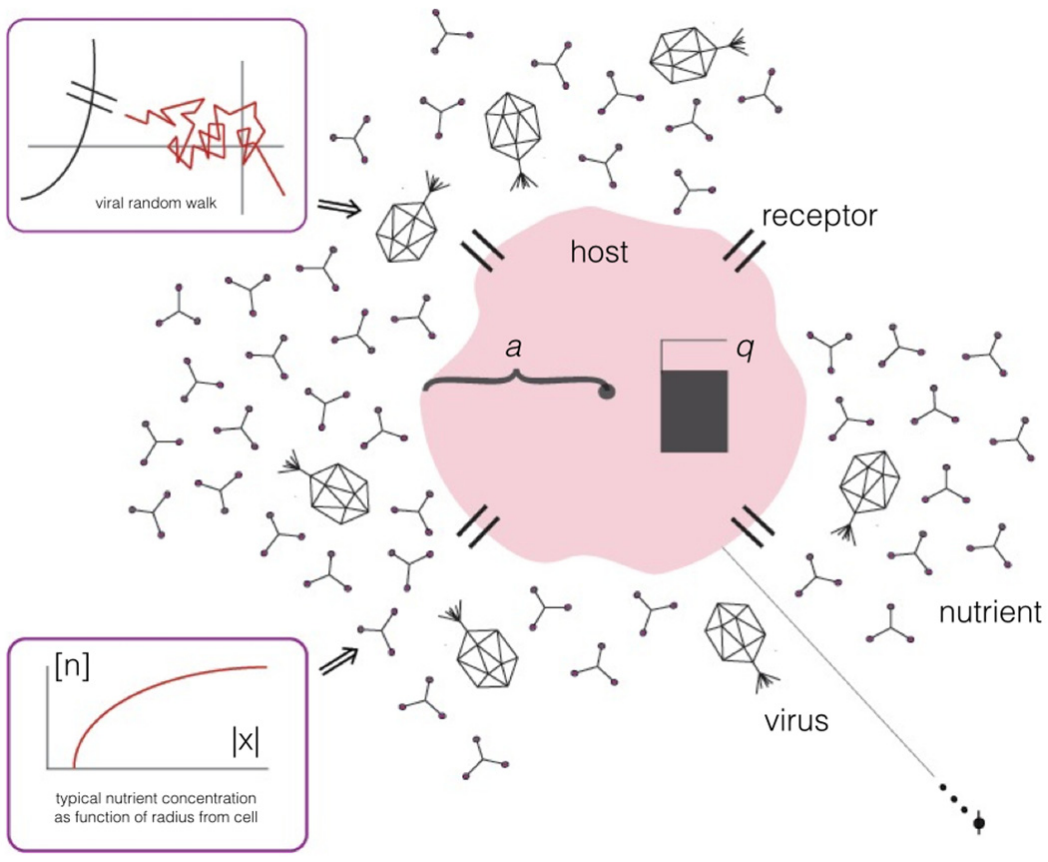
Model schematic. Viruses invade a would-be-host through the same receptor via which the host uptakes a limiting nutrient.

To investigate this problem from the perspective of an individual microorganism, we develop the simplest model that permits the essential dynamics. Consider a spherical, nonmotile microorganism, living in a typical marine environment with a given limiting nutrient, as well as a virus population which invades that organism through the receptors for said nutrient (Fig 1). Because we only consider one nutrient in the model, herein ‘co-limitation’ refers to simultaneous control by viruses and the limiting nutrient. This approach is in general applicable to any of a very large class of organisms living at small Sherwood number (i.e. that diffusive mass transfer dominates advective mass transfer) [28]; shape is unlikely to play a significant role in uptake dynamics at these scales [29], and as organisms are expected to relax their uptake rate to that where nutrients are co-limiting [30], the consideration of a single nutrient is not seriously restrictive. The case of multiple viruses may be interesting and relevant to real systems, but all of the expected results are well-treated in [21], and outside of the scope of this paper. In our parameter range selection we focus on marine systems, but the model should be equally applicable to other microbial systems as well.

**Fig 2:**
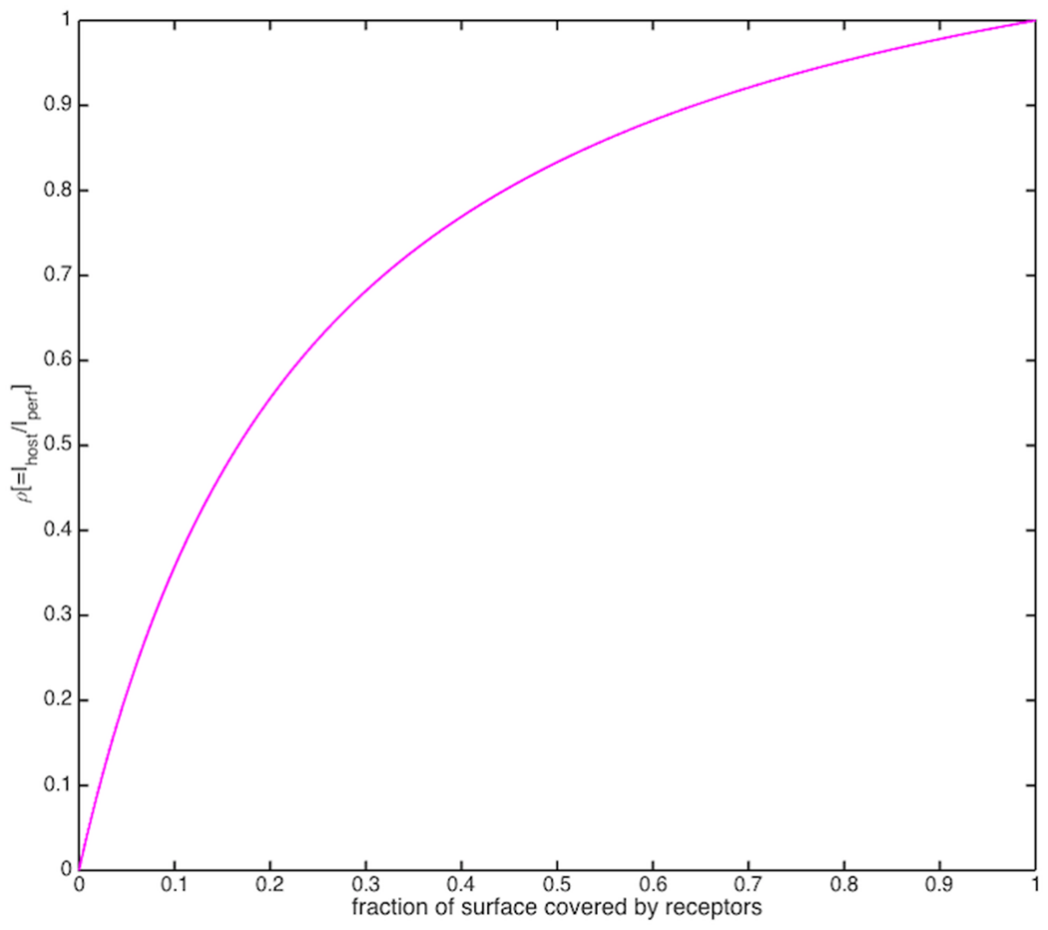
Plot of receptor efficiency versus percent receptor coverage. Similar to a Monod curve.

We track nutrient parameters with the subscript *ν* and virus parameters with the subscript *β*; host concentration will be tracked with the subscript *η*. Model parameters are then:

- concentration and diffusivity of nutrient and virus, resp. (*c*_*ν*_, *κ*_*ν*_, *c*_*β*_, *κ*_*β*_).
- host radius *a*, nutrient quota *q* (the mass of nutrient needed to uptake before replicating), and local concentration *c*_*η*_. As this is an individual model, by ‘local concentration’ we mean the concentration of hosts in a small region around the individual host, e.g. within a sphere centered at the host with radius *O*(10^−3^m); thus if hosts are aggregated or patchy, local concentration *c*_*η*_ increases.
- host receptor efficiency *ρ*, i.e. the ratio of the cell’s nutrient uptake rate with that of a perfectly absorbing sphere its size *I*/*I*_*perf*_, a monotonic function of the fraction of its cell surface covered by receptors (Fig 2) [31].

Table 1 displays observationally derived value ranges for the above parameters in various marine ecosystems [32–48]. Let nutrients be uptaken diffusively and let virsues travel in 3-d Brownian motion. Employing the canonical formula for nutrient uptake rate [31, 49] as a function of these parameters, *I* = 4*πρaκ*_*ν*_ *c*_*ν*_, a time-to-replication can easily be derived as the cell quota divided by the uptake rate, i.e. $\tau_{r}:=q/I$. As mentioned in the introduction, classically [25] virus uptake has been modeled as a diffusive absorption process as well, taken up in large numbers by hosts; however, marine viruses are known to be highly virulent [6, 17], thus it is not an unreasonable assumption to take it that a viral invasion corresponds to the eventual death of its host’s lineage; marine viral lysing timescales have been shown in many cases to be much shorter than host replication times (though are not always, e.g. for lysogenic viruses, for which this sort of model does not apply) [26, 27]. Thus we can model viral uptake probabilistically, with the virus taking a 3-dimensional random walk in a domain near its host, and deriving a probability density function for the first passage time to the host’s cell surface.

**Table 1:** Parameter definitions, typical values, and units.

parameter	definition/formula	typical range	units
	values taken from [12, 13, 25, 35–48] and sources compiled therein		
host radius (*a*_*η*_)	equivalent spherical radius [32]	10^−6^ − 10^−4^	m
nutrient radius (*a*_*ν*_)	”	10^−11^ − 10^−9^	m
viral radius (*a*_*β*_)	”	10^−8^ − 10^−7^	m
host concentration (*c*_*η*_)	local microscale concentration	10^6^ − 10^11^	m^−3^
nutrient concentration (*c*_*ν*_)	”	10^10^ − 10^14^	fg m^−3^
virus concentration (*c*_*β*_)	”	10^7^ − 10^12^	m^−3^
host nutrient quota (*q*)	mass of nutrient required to uptake before replicating	.1—10^6^	fg
host receptor efficiency (*ρ*)	fraction of uptake rate relative to perfect absorber, *I*/*I*_*perf*_	.1-1	-
nutrient diffusivity (*κ*_*ν*_)	Einstein-Stokes relation: $\kappa_{i}:=\frac{k_{B}T}{6\pi \nu a_{i}}$	10^−10^ − 10^−9^	m^2^/s
virus radial diffusivity ($\varkappa_{\beta }$)	projection of 3-d viral diffusivity onto radial vector, ∼.13*κ*_*β*_	10^−13^ − 10^−12^	m^2^/s
host replication timescale (*τ*_*r*_)	$\tau_{r}:=q/I=\frac{q}{4\pi \rho a_{\eta }\kappa_{\nu }c_{\nu }}$	<1/day—>1/week	s
host probability of replication (*P*_*r*_)	probability that *τ*_*r*_ < *τ*_*invasion*_	0—1	-
host lineage growth rate (*μ*)	expected growth rate incorporating virus-induced death	<0−>1/day	s^−1^

### Radial walk

**Fig 3:**
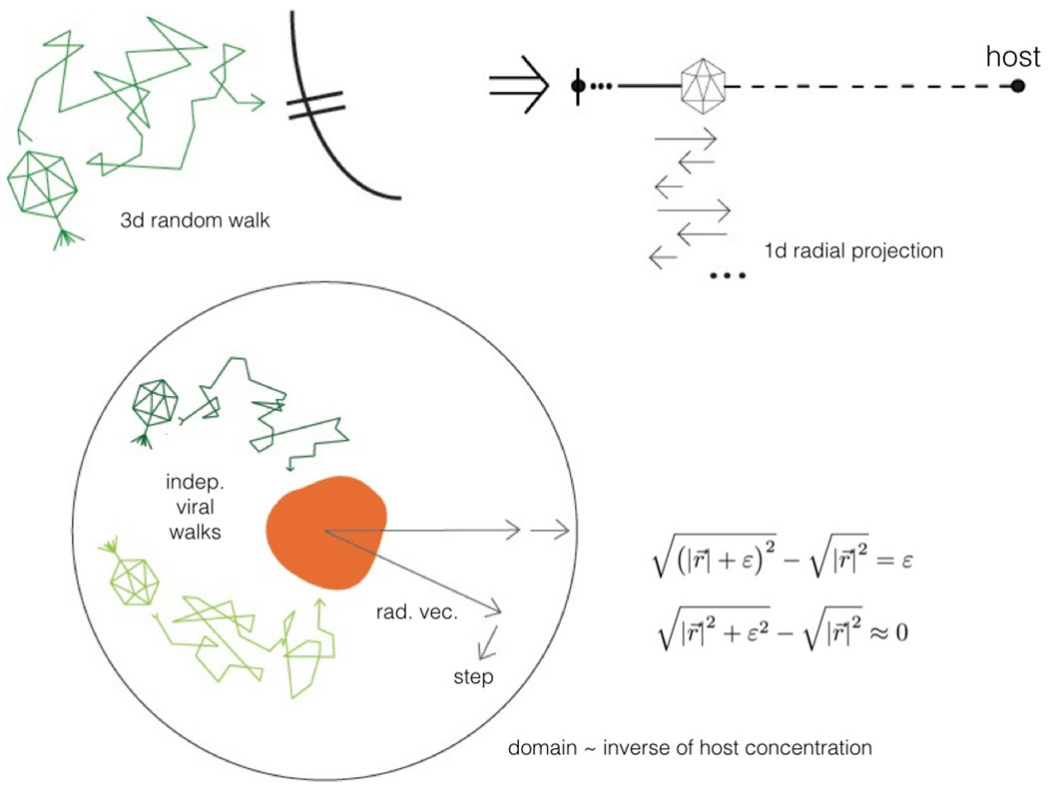
Viral walk projection schematic.

Since viral walks will be independent (as they are Brownian motion trajectories), consider a single virus randomly walking near a host, in spherical coordinates (*φ*, *ϑ*, *r*) with the origin at the host’s center; the angle coordinates are irrelevant for invasion, so optimally we’d like to project this to a 1-d walk. This in general is not possible, because spherical coordinates have curvature, but in this case we can take advantage of the fact that the mean step size for the virus is several orders of magnitude smaller than its radial coordinate vector, which is at least *O*(*a*) = *O*(*μ*m). When decomposing a given random step into its radial and nonradial components, the effect of the nonradial component on the radial distance after the step will be negligible; this is just by Pythagoras’ theorem. For a random step starting at radius *r* decomposed into radial part *s* and nonradial part *ε*, the new radial distance:

$$\begin{array}{c} \sqrt{{\left(r+s\right)}^{2}+\varepsilon^{2}}=\sqrt{r^{2}+2rs+s^{2}+\varepsilon^{2}}\approx \sqrt{r^{2}+2rs} \end{array}$$

even when *ε* ≫ *s* because r⋙s,ε. We thus can project the 3-d random walk onto a 1-d random walk with a redefined radial diffusivity $\varkappa_{\beta }$ (Fig 3), for which the orthogonal components can be incorporated as a negative drift; this drift is small enough for the parameter range of this model to be ignored; its inclusion has no impact on the results, and may even be surpassed by a positive drift towards host receptors caused by viruses actively seeking their hosts as thought possible in some cases [37]. To calculate $\varkappa_{\beta }$, we find the projected mean radial step length *δ*_1*d*_ for the given 3-d step length by integrating over all step directions, assuming that step angles are uncorrelated:

$$\begin{array}{ccc} \delta_{1d} & = & \delta_{3d}\int_{-1}^{1}\frac{\pi }{4}\cos(\frac{\pi }{2}r)\;dr=(1-\frac{2}{\pi })\delta_{3d}\\ & \Rightarrow & \varkappa_{\beta }:={\left(1-\frac{2}{\pi }\right)}^{2}\kappa_{\beta }\approx .13\kappa_{\beta } \end{array}$$

### First passage time

Chankdrasekhar [50, 51] showed that for randomly distributed particles in three dimensions the mean distance between particles of a given concentration *c* is $\frac{5}{9}c^{-1/3}$ (technically the prefactor is.554 ≈ 5/9); we may then consider the virus to be undergoing a 1-d radial random walk between *a* and $b:=\frac{5}{18}c_{\eta }^{-1/3}$, where we implement an absorbant boundary condition at *a* and a reflective/periodic boundary condition at *b*; the second boundary condition is included to account for the multiplicity of hosts and viruses in the local environment; a virus moving away from one host will be moving closer to another one nearby. We then may use the established result in probability theory that the time it takes for a virus starting at radial distance *γ* to hit the cell surface is given by a Lévy distribution *L* [52]; if we were to incorporate the small drift, we would use an Inverse Gaussian distribution, which has the Lévy distribution as its limit [53]. We then take this first passage probability for invasion time *τ*_*i*_ (without shifting from the origin, as is done in some cases):

$$\begin{array}{ccc} L(h;g^{2}) & := & \frac{g}{\sqrt{2\pi h^{3}}}\exp(-g^{2}/2h)\;\Rightarrow \;\\ 2P(\tau_{i}|\gamma ) & = & L\left(\tau ;\frac{{\left(\gamma -a\right)}^{2}}{\varkappa_{\beta }}\right)+L\left(\tau ;\frac{{\left(2b-a-\gamma \right)}^{2}}{\varkappa_{\beta }}\right) \end{array}$$

where we incorporate the possibility that the virus travels all the way to the reflective boundary and back to the cell surface, or equivalently travels away from another cell’s and to the surface of that host, and normalize accordingly, as we have added two probability distributions. We can then find the probability of time-to-invasion by integrating over all possibile initial positions for the virus in the dining sphere, then define probability *p*_*r*_ that *τ*_*r*_ < *τ*_*i*_, i.e. that a host starting with a single virus in its dining sphere successfully replicates. Assuming our times are sufficiently small that $\left(b-a\right)/\left(\sqrt{\varkappa_{\beta }\tau }\right)\geq 2$ (which is more than guaranteed by host replication timescales being on the order of a day) then dropping small terms, this expression simplifies to:

$$\begin{array}{c} p_{r}=1-\int_{o}^{\tau_{r}}E_{\gamma }\left\{P\left(\tau_{i}|\gamma \right)P\left(\gamma \right)\right\}\;d\tau_{i}\approx 1-\frac{4}{\pi }\frac{a^{2}}{b^{3}-a^{3}}\sqrt{\varkappa_{\beta }\tau_{r}} \end{array}$$

These results are well-matched by a similar derivation one can take using electrostatics [53] and by the CDF of the Lévy distribution which is a complimentary error function, but more instructive in that the above expression reveals direct parameter dependencies and provides additional intuition as to the importance of various terms than an expression involving modified Bessel functions or erfc(⋅).

### Growth rate

**Fig 4:**
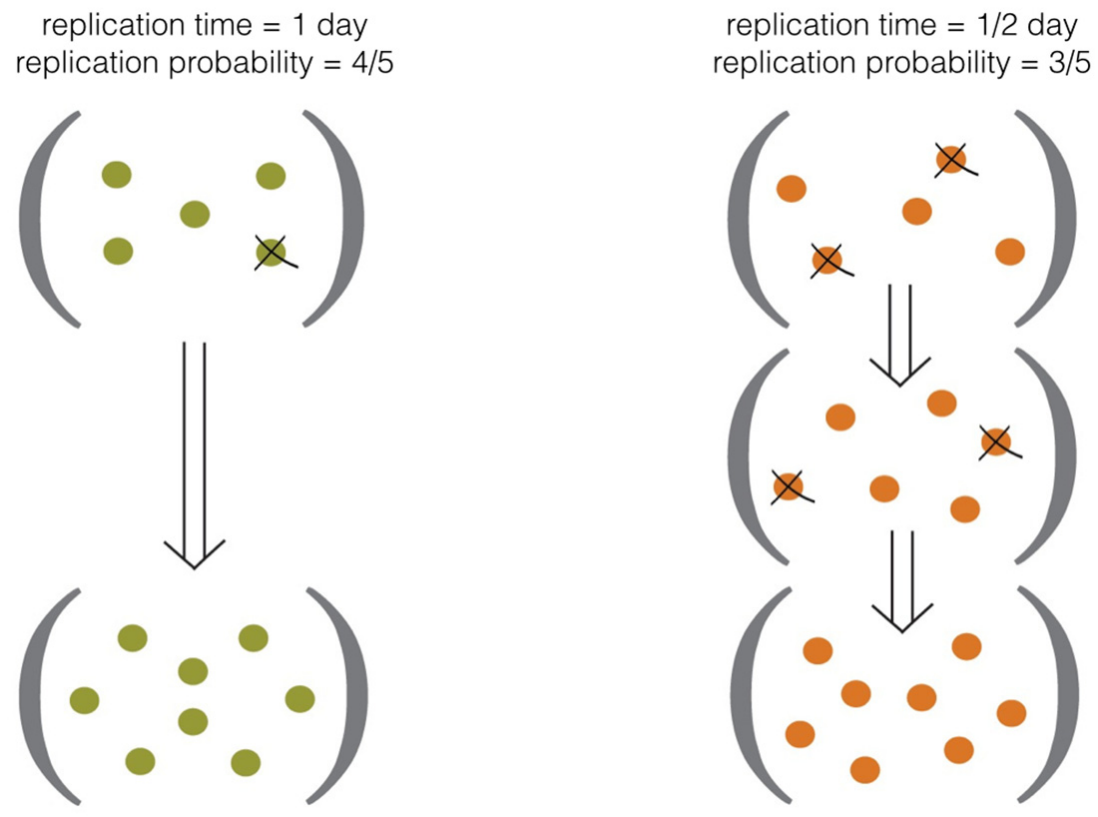
Schematic of individual lineage virus-modified growth rate *μ*. success is determined both by viral susceptibility and replication rate.

However, this above expression doesn’t tell the whole story; ultimately the most successful strategy for a lineage is determined by a combination of this replication probability, the number of viruses in play, and the *time* it takes to replicate, because replication is a compounding process; a faster, riskier strategy can beat out a more conservative strategy (Fig 4). Ultimately, then, the desired parameter is the growth rate. If we consider a replication event as a doubling, and there are *n*_*β*_ viruses in the host’s dining sphere, we can define the (expected, individual, viral-uptake-included) growth rate *μ* as [54]

$$\begin{array}{c} \mu :=\ln({\left(2p_{r}^{n_{\beta }}\right)}^{1/\tau_{r}})=\frac{1}{\tau_{r}}(\ln2+n_{\beta }\ln p_{r}) \end{array}$$

Noting that $n_{\beta }=\frac{4\pi }{3}\rho c_{\beta }b^{3}=\frac{5^{3}\pi }{2\cdot 3^{7}}\rho c_{\beta }/c_{\eta }\approx .089\rho c_{\beta }/c_{\eta }$ because viral trajectories are independent and the likelihood of a virus successfully contacting a receptor (invasion site) is proportional to the host’s receptor efficiency by construction, and plugging in our previously derived formulae for *p*_*r*_ and *τ*_*r*_, we find:

$$\begin{array}{c} \mu =\frac{4\pi \kappa_{\nu }c_{\nu }a_{\eta }\rho }{q}(.69+.089\rho \frac{c_{\beta }}{c_{\eta }}\ln(1-.13[\frac{a_{\eta }^{3/2}}{{\left(\frac{5}{18}\right)}^{3}c_{\eta }^{-1}-a_{\eta }^{3}}]\sqrt{\frac{a_{\nu }q}{a_{\beta }c_{\nu }\rho }}))\;\left(*\right) \end{array}$$

where we have taken *κ*_*β*_/*κ*_*ν*_ = *a*_*ν*_/*a*_*β*_ as the ratio of their radii, derived from the Einstein-Stokes relation $\kappa_{i}:=\frac{k_{B}T}{6\pi \nu a_{i}}$ [29], and added the subscript *η* to the host’s radius; the above equation is the same if *a*_*ν*_/*a*_*β*_ is replaced by *κ*_*β*_/*κ*_*ν*_. Our desired expression for the individual host lineage growth rate in the presence of both nutrients and viruses. Freezing parameters other than *ρ*, this equation takes the form

$$\begin{array}{c} \mu =A\rho +B\rho^{2}\ln\left(1-C\rho^{-1/2}\right)\;\left(**\right) \end{array}$$

$$\begin{array}{c} A:=4\pi \ln2\kappa_{\nu }c_{\nu }a_{\eta }/q\;B:=.13A\frac{c_{\beta }}{c_{\eta }}\;C:=.13[\frac{a_{\eta }^{3/2}}{{\left(\frac{5}{18}\right)}^{3}c_{\eta }^{-1}-a_{\eta }^{3}}]\sqrt{\frac{a_{\nu }q}{a_{\beta }c_{\nu }}} \end{array}$$

Where *μ*, *A*, and *B* have units of inverse time, and *C* is dimensionless. The recurrence of the prefactor.13 is a coincidence, as would be clear if we expanded past two significant digits. The term *Aρ* indicates the standard “virusless” growth rate, and the always-negative second term indicates the impact of viral invasion on growth rate, which is affected not only by viral parameters but by host and nutrient parameters as well, reflecting the importance of invasion as well as replication timescales in the growth rate.

## Results

The above expression provides a framework to investigate sensitivity of growth rate to different parameters, and more interestingly to suggest under what conditions growth rate might be optimized with respect to receptor coverage. After plugging in ranges of typical values for parameters as suggested in Table 1, we found:

- *μ* decreases linearly as virus concentration *c*_*β*_ increases, as expected, because more viruses are present to increase probability of invasion. *μ* increases superlinearly in nutrient concentration *c*_*ν*_, because not only is the virus-free growth rate increasing linearly but the probability that *τ*_*r*_ < *τ*_*i*_ is increasing simultaneously.
- diffusivities may change because of changing temperature or different radii of the diffusing particle; higher diffusivities will result in higher encounter rates. If temperature increases, *μ* increases linearly because the difference in *κ*_*β*_ is offset by proportional increase in *κ*_*ν*_ in the coefficient *C*, while the coefficient *A* ∝ *κ*_*ν*_; if diffusivities change because of changes in radii, *a*_*ν*_ decreasing will cause *μ* to increase superlinearly, for similar reasons to the effect of *c*_*ν*_ above, while a decrease in *a*_*β*_ will cause *μ* to decrease.
- *μ* and host nutrient quota *q* are approximately inversely proportional, as expected, because *τ*_*r*_ ∝ *q*.
- *μ* decreases superlinearly as host radius *a*_*η*_ increases, as can be seen most clearly in the expression for *p*_*r*_; this is consistent with classical theory in viral dynamics [26], though derived quite differently.

The more interesting behavior is that of receptor coverage *ρ* and host concentration *c*_*η*_; if *A* ≫ *B* in (**) above, *c*_*η*_ has negligible effect and *μ* increases linearly with *ρ*. However, in cases of $.089\frac{c_{\beta }}{c_{\eta }}\geq O\left(1\right)$, we have *O*(*A*) = *O*(*B*), and hosts may optimize *μ* with respect to *ρ*. Because methods of estimating ocean viral concentrations do not in general differentiate between viral types, but do almost always register higher concentrations of viruses than phytoplankton or microbes in the ocean, it is difficult to get good estimates of *c*_*β*_, but it is known that the value of *c*_*β*_/*c*_*η*_ may range over several orders of magnitude but can often be ≥1; on average we might expect *B*/*A* = 1.3 if on average *c*_*β*_ ≈ 10*c*_*η*_ [14].

**Fig 5:**
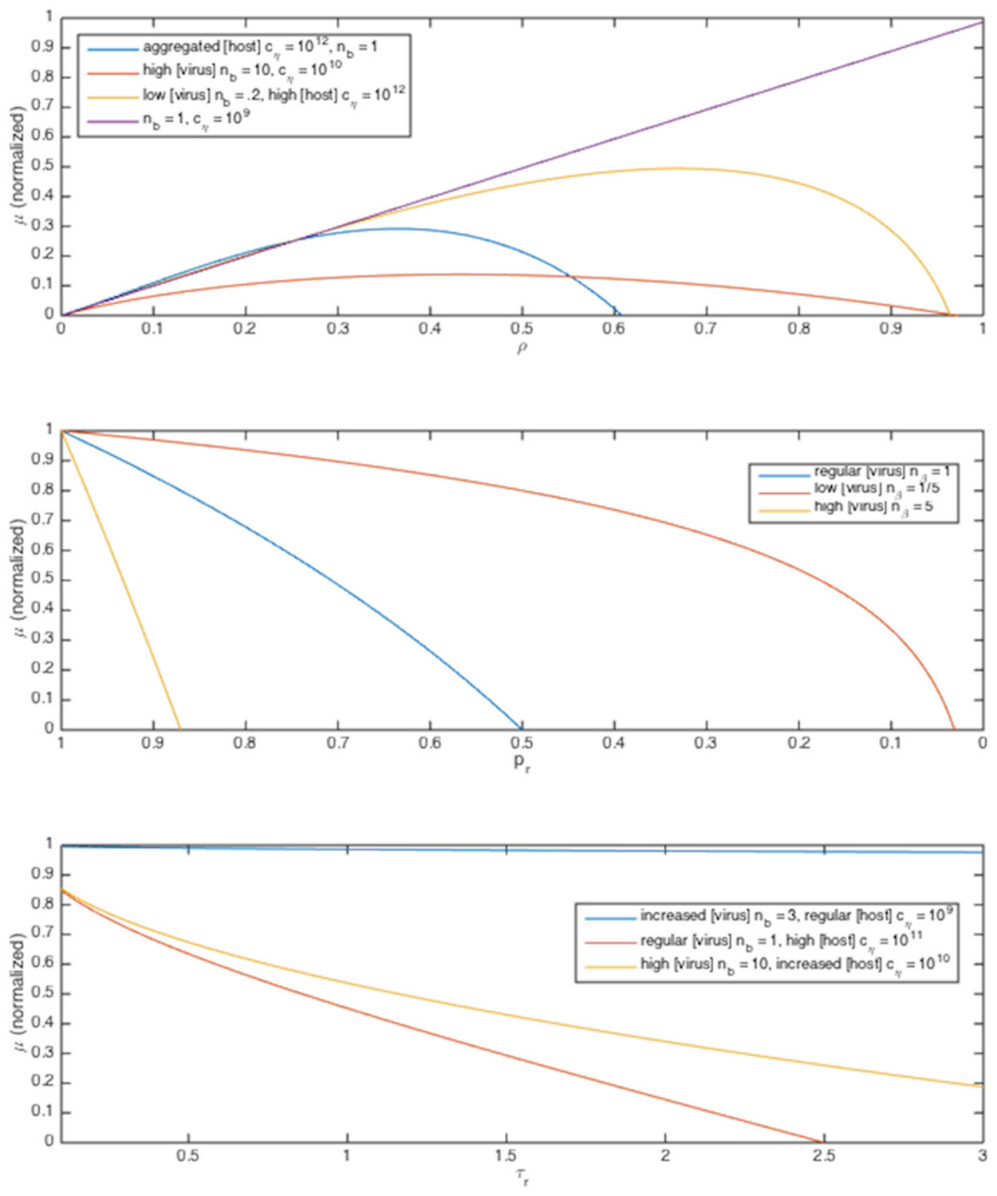
Growth rate *μ* as a function of several parameters. A:*μ* as a function of *ρ* in Regimes I-III. Parameter values given in legends.

Even if the bulk concentration of hosts may be low, as the individual in this case only is affected by the *local* concentration at its own scale, *c*_*η*_ may be significantly larger than if hosts were uniformly distributed, which can significantly change the value of *C*, which may thus range over several orders of magnitude dependent on the various parameter values [55, 56]. Thus the model suggests that in cases where the concentration of viruses is at least an order of magnitude than that of its hosts, or the hosts are aggregated, that optimal growth rate may be achieved with *ρ* lower than 1 (Fig 5). After exploring the observationally suggested parameter space, we can simplify to four regimes:

Regime I: If *A* ≫ *B* and *C* is not particularly large, *μ* ≈ *Aρ*, this is maximized for *ρ* = 1. That is, if viruses are not particularly locally abundant relative to hosts, and hosts are not particularly large or abundant, an individual lineage’s growth rate will be unaffected by viruses, as we might expect; this does not, however, imply viral invasion rates are non-negligible for the population.

Regime II: If *A* ≤ *B*, and *C* is not particularly small, for large range of values for *C*, there will be an optimum in *μ* with respect to *ρ*. That is, if viruses are locally abundant relative to hosts, individual lineages’ growth rates can be optimized with respect to receptor coverage, i.e. hosts with more conservative uptake rates may be more successful.

Regime III: If *A* > *B* but *C* = *O*(1) or larger, there will be an optimum in *μ* with respect to *ρ*. That is, if hosts are at high local concentrations, whether in bloom or aggregated, especially for larger hosts, their individual risk for viral invasion increases, because if an individual virus enters the region of aggregation its likelihood of contacting a host increases significantly. This carries the implicit assumption that host aggregation is un- or positively correlated with viral aggregation, which is difficult to measure on microscales, but plausible; in a patch of hosts there is an increased likelihood that viruses are present in at least as significant numbers because of an increased likelihood that a lysing event has recently occurred or will soon occur, releasing a large number of viruses. This colocation is a subtle component needing further consideration.

Regime IV: In some cases, parameters *C* and/or *B* can become large enough that *μ* is negative for nearly all *ρ* values; in these cases, the viral invasion rate is high enough that the lineage of a given individual is unlikely to survive, so the population is grazed by viruses down to a small seed population.

## Discussion

**Fig 6:**
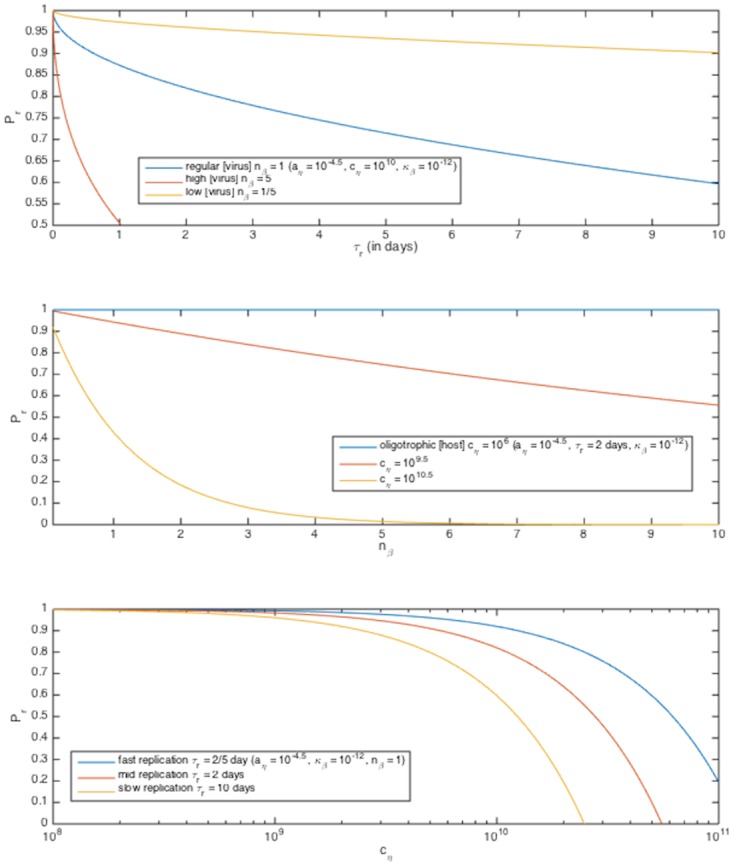
Total replication probability *P*_*r*_ as a function of several parameters. Parameter values given in legends.

The above model investigates the impact of simultaneous bottom-up and top-down control on growth rate of generalized microbes, from an individual-mechanistic perspective. From it we can derive the expected probability of successful individual replication *P*_*r*_ (Fig 6) as a function of host radius *a*_*η*_, host concentration *c*_*η*_, viral diffusivity *κ*_*β*_, number of viruses nearby *n*_*β*_, and host replication timescale *τ*_*r*_:

$$\begin{array}{c} P_{r}={\left(p_{r}\right)}^{n_{\beta }}={\left(1-\frac{.93\;a_{\eta }^{2}\sqrt{\kappa_{\beta }\tau_{r}}}{{\left(\frac{5}{18}\right)}^{3}c_{\eta }^{-1}-a_{\eta }^{3}}\right)}^{n_{\beta }} \end{array}$$

as well as an expression for a modified, individual lineage growth rate incorporating both nutrient uptake limitation and expected viral invasion limitation as a function of either the parameters used above or our initially described parameters. It suggests that receptor efficiency *ρ* and host aggregation are key factors for understanding the dynamics involved, in addition to host, nutrient, and virus concentrations. Both expressions yield intuitively plausible parameter dependencies.

Beyond corroborating the findings of the studies which contextualize it, the model also suggests that co-limitation exerts influence on organisms at shorter timescales, i.e. those on the order of several doubling times of an organism’s lineage. We can draw several other suggestions from the model:

- Cases where organisms feel selection pressure to optimize towards co-limitation may only be felt in specific parameter ranges where concentrations of relevant viruses are significant, or hosts are tightly packed, subsequently increasing viral invasion risk—otherwise, receptor coverage *ρ* will be determined by other, likely biochemical, factors. However, this model does suggest that this selection pressure can be felt at the individual scale, bridging the gap with previously suggested population-scale selection pressure. Both of these model implications are difficult to justify without mechanistic treatment presented here.
- Intermittency of viral concentration experienced by a population may be a process that sustains phenotypic diversity of receptor coverage, because different *ρ*-valued phenotypes will have superior growth rates in different viral conditions.
- Organisms which are capable of changing their active receptor coverage may be strongly advantaged by optimizing growth rate with respect to *ρ* as conditions fluctuate. Even if these organisms cannot detect cues that viruses are present, other cues such as infochemicals from nearby conspecifics (indicating aggregation) or increases in local nutrient concentration (because presumably nutrient concentration will be correlated with host aggregation) may be used to, perhaps counterintuitively, *decrease* *ρ* values so as to maximize growth rate by minimizing risk of viral invasion.
- The model suggests that viral top-down control can exert selective influence on populations at very short timescales, via differential grazing; parasite-host models often consider bottom-up factors to drive selection on long timescales, so this suggests a plausible disjoint range of times on which top-down pressure can still be significant [57].
- As the above function for *μ* is very sensitive to parameters inside the logarithm, i.e. *C*, it indicates that in logarithmic space there is a relatively narrow range where viruses can both exert influence on host growth and not kill off the bulk of the population. This range largely overlaps with ranges found for oceanic viruses, which may to a limited extent explain the magnitude of their distribution in marine environments.

While co-limitation by multiple nutrients is common in many ecosystems [30, 58, 59, 60], population co-limitation does not equate to co-limitation of individuals [30], and the impact of viruses invading through a particular nutrient’s receptor can only serve to drive down or keep the same the receptor coverage for that nutrient; thus it is unclear that considering multiple nutrients in the model above would allow the gleaning of any new results or additional relevance. However, it may be interesting to investigate the impact of motility or shear, i.e. increasing Sherwood number, on the model, or the impact of viral mortality; note that in the above model the viruses live forever, but a possibility of viral death modifying the Lévy distribution might change the above expression for *P*_*r*_. Other factors worth further consideration that may intersect with the model could be the impact of Michaelis-Menten kinetics, relationships of replication and invasion timescales with invsasion-to-lysing timescales, and mechanisms which may increase or decrease the colocation of viruses, hosts, and nutrients.

The model herein suggests many possible complex virus-nutrient-host interrelationships, worthy of further investigation, both empirical and theoretical. Rigorously understanding the influence of viruses on marine populations remains an intricate and important problem.

## Supporting Information

S1 File Supplementary Data. Data as reported in table, taken from references, used to determine ranges of model parameters, as a.csv file.
(CSV)
